# Freshwater Ammonia-Oxidizing Archaea Retain *amoA* mRNA and 16S rRNA during Ammonia Starvation

**DOI:** 10.3390/life5021396

**Published:** 2015-05-19

**Authors:** Elizabeth French, Annette Bollmann

**Affiliations:** Department of Microbiology, Miami University, 32 Pearson Hall, 700 East High Street, Oxford, OH 45056, USA; E-Mail: frenche2@miamioh.edu

**Keywords:** nitrification, ammonia oxidation, ammonia-oxidizing archaea, ammonia-oxidizing bacteria, starvation, ammonia monooxygenase

## Abstract

In their natural habitats, microorganisms are often exposed to periods of starvation if their substrates for energy generation or other nutrients are limiting. Many microorganisms have developed strategies to adapt to fluctuating nutrients and long-term starvation. In the environment, ammonia oxidizers have to compete with many different organisms for ammonium and are often exposed to long periods of ammonium starvation. We investigated the effect of ammonium starvation on ammonia-oxidizing archaea (AOA) and bacteria (AOB) enriched from freshwater lake sediments. Both AOA and AOB were able to recover even after almost two months of starvation; however, the recovery time differed. AOA and AOB retained their 16S rRNA (ribosomes) throughout the complete starvation period. The AOA retained also a small portion of the mRNA of the ammonia monooxygenase subunit A (*amoA*) for the complete starvation period. However, after 10 days, no *amoA* mRNA was detected anymore in the AOB. These results indicate that AOA and AOB are able to survive longer periods of starvation, but might utilize different strategies.

## 1. Introduction

In natural systems, the majority of microorganisms exist in a physiological state similar to a stationary phase [1]. Nutrients become available in short pulses or are present in low concentrations. To survive these periods of starvation, microbes must be able to respond rapidly to fresh nutrients when they become available. Ammonium is one of the nutrients that is often limiting due to low nitrogen input and/or competition with other microorganisms.

Ammonia-oxidizing archaea and bacteria (AOA and AOB) are two groups of organisms that often encounter substrate starvation in the environment. Both AOB and AOA use the oxidation of ammonia to nitrite for the generation of energy. In AOB, this process is catalyzed by the enzymes ammonia monooxygenase (Amo) and hydroxylamine oxidoreductase (Hao) [2]. The first step of ammonia oxidation in AOA is also catalyzed by Amo; however, the subsequent steps of the reaction remain uncharacterized [3]. AOB exhibit strain-specific differences in their affinity for ammonia and the ability to compete for ammonia, but they share the ability to withstand extended periods of ammonia starvation [4,5,6,7,8,9,10,11]. The mechanism of ammonia starvation in AOB has not been fully elucidated; however, previous work demonstrated that *amoA* mRNA and 16S rRNA stability, as well as the stability of the Amo protein, during starvation may play a critical role in their survival and rapid recovery [4,7,12]. AOA have a higher affinity for ammonium than AOB [13,14]. *Nitrosopumilus maritimus* keeps a low number of *amoA* transcripts present in the cell during short-term starvation [15]. However, it is still unknown how quickly AOA recover from starvation and what happens to the ribosomes during starvation.

Here, we present experiments to monitor the starvation and recovery of the AOA enrichment culture AOA-AC1 and the AOB enrichment culture AOB-G5-7. We investigated the recovery time after starvation and the levels of *amoA* mRNA and 16S rRNA during starvation for a period up to almost two months.

## 2. Results

Both cultures (AOA-AC1 and AOB-G5-7) were inoculated in triplicate into mineral salts medium with 0.5 mM ammonium. During growth, ammonium was converted to nitrite/nitrate (Figure S1). The specific growth rates were 0.017 ± 0.0004 h^−1^ (AOA-AC1) and 0.057 ± 0.018 h^−1^ (AOB-G5-7) before the cultures entered starvation. Starvation started when ammonium was no longer detectable in the cultures (Figure S1). Samples from the cultures at the end of the growth phase and during starvation were inoculated into fresh mineral salts medium with 0.5 mM ammonium (recovery cultures); nitrite/nitrate was measured, and the specific growth rate and lag phase were determined (Figure S2).

**Figure 1:**
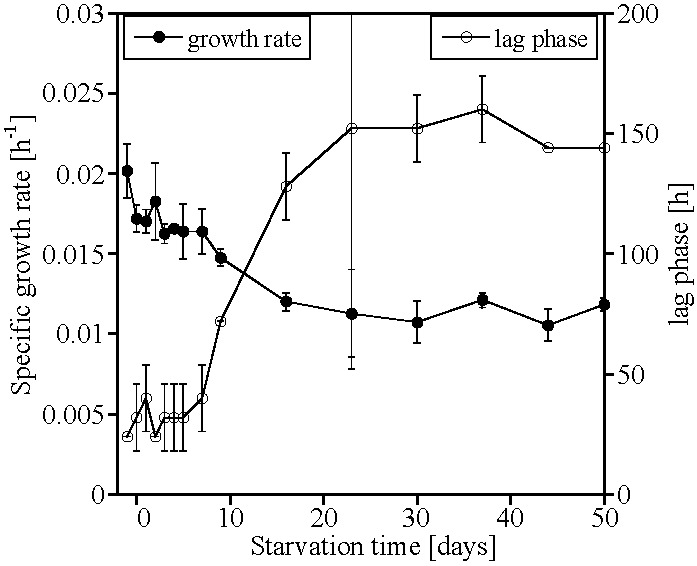
Specific growth rate (h^−1^) and lag phase (h) of the recovery cultures after starvation of the ammonia-oxidizing archaea (AOA) enrichment culture AOA-AC1 (mean ± SD, *n* = 3). Starvation started at Day 0.

The specific growth rates of the recovery cultures of AOA-AC1 did not differ significantly during the first ten days of starvation from those generated with late logarithmic/early stationary phase cultures. Recovery cultures inoculated with cultures that had been starved for 10 days or longer had a significantly longer lag phase than those inoculated with early stationary phase cultures. The specific growth rates of AOA-AC1 decreased after Day 10 of starvation, reaching a stable, lower value around Day 20 of starvation (Figure 1, Table S1 and Table S2).

**Figure 2:**
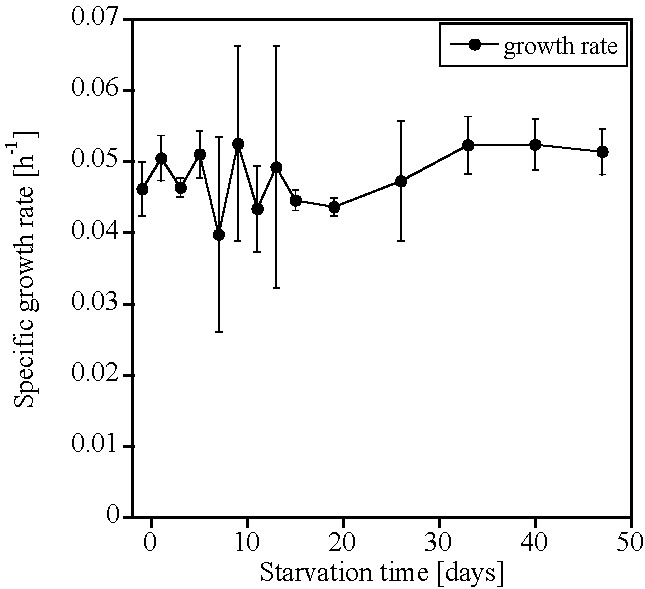
Specific growth rate (h^−1^) of the recovery cultures after starvation of the ammonia-oxidizing bacteria (AOB) enrichment culture AOB-G5-7 (mean ± SD, *n* = 3). Starvation started at Day 0. The lag phase of AOB-G5-7 was always 24 h or less, because the first sample from the recovery cultures was taken after 24 h, and the culture was always active at that time.

No significant differences were observed in the specific growth rate of any AOB-G5-7 recovery cultures, regardless of the length of the starvation period (0–50 days; Figure 2, Table S1).

RNA abundance (16S rRNA and *amoA* mRNA of AOA-AC1 and AOB-G5-7 and bacterial 16S rRNA of all bacteria in the cultures) was determined in selected samples over the course of the starvation period. The RNA concentration in the samples did not significantly change in the AOA-AC1 cultures, while it increased slightly in AOB-G5-7 (Table S3).

**Figure 3:**
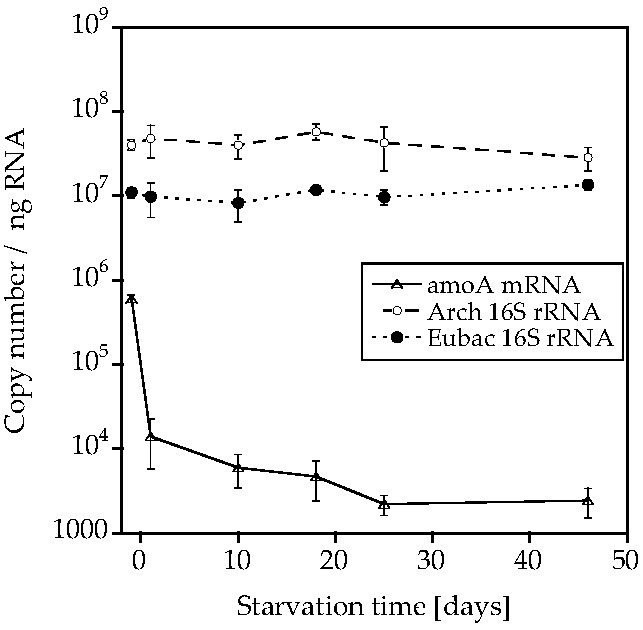
Archaeal *amoA* mRNA, AOA-specific 16S rRNA and bacterial 16S rRNA abundance (copies/ng RNA) during starvation of the AOA enrichment culture AC1 (mean ± SD, *n* = 3).

**Figure 4:**
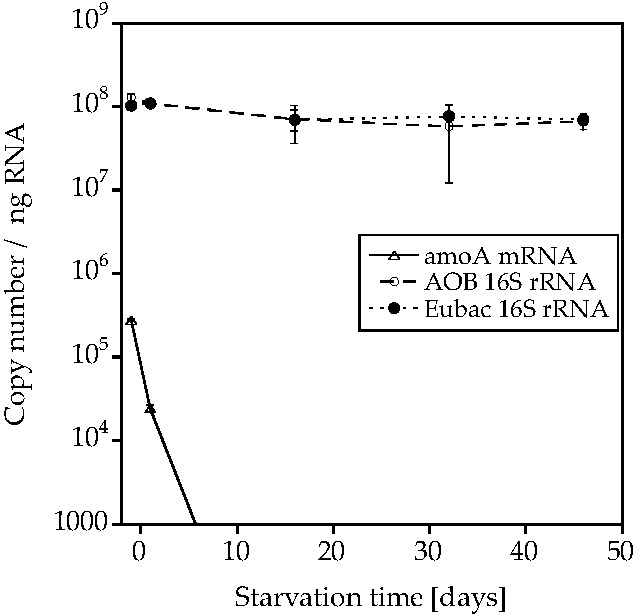
Bacterial *amoA* mRNA, AOB-specific 16S rRNA and bacterial 16S rRNA abundance (copies/ng RNA) during starvation of the AOB enrichment culture AOB-G5-7 (mean ± SD, *n* = 3).

AOA-AC1 retained both *amoA* mRNA and 16S rRNA, while AOB-G5-7 only retained the 16S rRNA (Figure 3 and Figure 4; Table S4, Table S5 and Table S6). The *amoA* mRNA abundance decreased by two orders of magnitude in AOA-AC1 after 10 days of starvation and stayed constant around 1000–10,000 copies/ng RNA for the rest of the starvation period. The 16S rRNA abundance specific for AOA or AOB decreased slightly, but not significantly in the respective cultures. The bacterial 16S rRNA abundance decreased slightly in AOB-G5-7, but stayed constant in AOA-AC1.

## 3. Discussion

AOA-AC1 and AOB-G5-7 both recovered ammonia-oxidizing activity after up to 50 days of starvation; however, AOB-G5-7 recovered quicker than AOA-AC1 (Figure 1 and Figure 2). Both cultures retained 16S rRNA (ribosomes) during the starvation period (Figure 3 and Figure 4). Similar observations were made for other AOB, such as *Nitrosospira briensis* [4] and *Nitrosomonas cryotolerans* [6,7]. These results indicate that ribosome retention is very likely a shared adaptive strategy of AOA and AOB to survive nutrient starvation for long periods of time.

Interestingly, AOA-AC1 and AOB-G5-7 behaved differently with respect to *amoA* retention. The *amoA* mRNA in the AOA cultures was detected during the complete starvation period, while *amoA* mRNA in the AOB culture was only detected during the first few days. This difference could give insight into the differences in the starvation recovery strategies of AOA and AOB. During the initial starvation period, AOA-AC1 recovered quickly from starvation, but after 10 days, the recovery period increased. At the same time, the number of *amoA* mRNA copies decreased from 10^6^ to 1000 copies/ng RNA and stayed constant at that level for the rest of the starvation period. The ammonia oxidizing activity of starved *Nitrosospira briensis* recovered much slower after treatment of the cells with acetylene, an irreversible inhibitor of the ammonia monooxygenase, than in untreated cells, indicating that treated cells had to synthesize new protein, while untreated cells were able to directly oxidize ammonia with the existing Amo protein [4]. Immunoblot analysis showed long-term stability and an increase in abundance of the AmoB protein in *Nitrosomonas eutropha* during a one-year starvation period [12]. Therefore, the stability of the Amo protein complex is very likely to play an important role in the recovery of the AOB after starvation. AOA-AC1; however, it could have another mechanism, because the cells retain *amoA* mRNA for a longer period, and their recovery patterns show slowed down recovery. Unfortunately, we do not know anything about the protein stability in AOA and can therefore only speculate about the recovery mechanisms in AOA. Since the length of the recovery of the AOA increases over time, the Amo enzyme might be degraded and forcing the cells to synthesize new enzyme, while their mRNA might be constitutively expressed. Constitutive expression of genes involved in nitrogen metabolism has been observed in the oligotrophic marine microorganism *Candidatus* Pelagibacter ubique under nitrogen starvation [16]. To elucidate the mechanisms in more detail, future studies could compare mRNA and protein stability in short- and long-term starved AOA and AOB.

## 4. Conclusions

The cultures AOA-AC1 and AOB-G5-7 show differences in the retention of the *amoA* mRNA, as well as in the recovery of the ammonia-oxidizing activity. These differences indicate that the two microbes might use different strategies to survive longer periods of starvation.

## 5. Material and Methods

Cultures, AOB-G5-7: We used the AOB freshwater enrichment culture G5-7 that was enriched from Lake Drontermeer in the Netherlands (AOB-G5-7) as representative AOB culture. This culture contains the AOB *Nitrosomonas* sp. Is79, a close relative of *Nitrosomonas oligotropha*, with 97.8% identity on the 16S rRNA gene level, a nitrite oxidizer and co-cultivated heterotrophic bacteria and grows well at ammonium concentrations between 0.25 mM and 5mM [10,17]. AOA-AC1: The enrichment culture AOA-AC1 was enriched from the sediment of Lake Acton (Ohio) and contained an AOA that was 99% identical based on the *amoA* sequence and 99.7% based on the 16S rRNA gene sequence to the AOA in the enrichment culture AOA-DW belonging to the water column/sediment Group I.1a of the Thaumarchaeota [18,19], a nitrite oxidizer and co-cultivated heterotrophic bacteria. The culture was around 80% enriched and contained no PCR-detectable AOB. Growth experiments showed that AOA-AC1 behaves similarly to the previously characterized AOA enrichment cultures AOA-DW, AOA-AC2 and AOA-AC5 [18,19]. AOA-AC1 grew at ammonium concentrations between 0.05 mM and 1 mM [19].

Medium: Mineral salts medium (MS medium) was used in all experiments containing 0.5 mM ammonium as (NH_4_^+^)_2_SO_4_, 10 mM NaCl, 1 mM KCl, 1 mM CaCl_2_∙2H_2_O, 0.2 mM MgSO_4_∙7H_2_O, 1 mL L^−1^ trace elements solution and 2 mM HEPES [20]. The pH was adjusted to 7.5 before autoclaving with 1 N NaOH. After autoclaving, separately-autoclaved KH_2_PO_4_ solution was added to obtain a final concentration of 0.4 mM [20].

Starvation experiment: The enrichment cultures AOA-AC1 and AOB-G5-7 were inoculated in triplicate using 10% v/v into 1 L mineral salts medium with 0.5 mM ammonium in 2-L Erlenmeyer flasks and incubated at 27 °C in the dark. The ammonium and nitrite/nitrate concentrations were measured on a daily basis to determine the onset of the starvation period. When the cultures had consumed approximately 80% of the initial ammonium concentration, they were assumed to be in the late logarithmic phase, and the first sample was removed for recovery experiments and molecular analysis. Once the ammonium in the 1-L cultures was completely consumed, the cultures were assumed to be starving. After this point, samples were collected for nitrite/nitrate and ammonium determination and recovery cultures every 24–48 hours for ten days and then once per week until the end of the experiment. Samples for molecular analysis were taken once per week.

Recovery cultures: Five mL of the cultures were inoculated into 45 mL of fresh mineral salts medium with 0.5 mM ammonium in a 125-mL Erlenmeyer flask to generate recovery cultures. The recovery cultures were incubated at 27 °C in the dark and sampled on a daily basis to determine the nitrite/nitrate concentration. The specific growth rates were calculated from the linear increase of the log-transformed nitrite/nitrate concentrations over time, assuming that nitrite/nitrate production in the cultures is correlated with the growth of AOA and AOB [13,18,20,21].

Chemical analysis: Ammonium, nitrite and nitrate concentrations were determined in cell-free supernatants using colorimetric methods [20,22,23,24].

RNA isolation and conversion into cDNA (starvation experiment): Samples of the starved cultures were collected for molecular analysis by filtering 50 mL onto 0.1-µm polycarbonate filters (AOA-AC1) and 0.22-µm nitrocellulose filters (AOB-G5-7). RNA was isolated from the nitrocellulose filters using the Qiagen RNeasy RNA kit (Qiagen, Valencia, CA, USA) according to the manufacturer’s recommendations with the following modifications. Acid-washed zirconium beads (1 g) (BioSpec Products, Bartlesville, OK, USA) and 1 mL buffer RTL were added to the filters. The filters were homogenized by bead beating for 30 s at 4800 rpm and shaking the tubes on a titer plate shaker for 10 min at the highest speed. The RNA was eluted with 30 µL RNase-free water. The RNA was treated with Ambion DNaseI (Life Technologies, Grand Island, NY, USA) for 60 min at 37 °C. RNA concentration and quality were measured by NanoDrop^TM^ 3300 Fluorospectrometer (Thermo Fisher Scientific, Wilmington, DE, USA). cDNA was synthesized using the Promega GoScript Reverse Transcriptase System (Promega) with random primers in 10 µL volumes with 2 µL samples.

Quantitative PCR (qPCR): The copy numbers of the 16S rRNA and *amoA* transcripts (cDNA and RNA) were quantified using the Bioline SensiFAST SYBR NoROX Kit (Bioline USA, Taunton, MA, USA) in 5-µL reactions with 0.5 µL sample in an Illumina Eco Real-Time PCR system (Illumina, San Diego, CA, USA) with the primers and conditions presented in Table S7 and Table S8. Primers were designed with the NCBI-primer-BLAST tool or used as published (Table S7) [25,26]. All primers were tested for specificity to the respective DNA. Standard curves were constructed using plasmids containing the gene sequence of interest. The efficiency of the reactions ranged from 94% to 104% (Table S2). R^2^ was in all experiments >0.99. To determine the copy numbers in the RNA samples, qPCR was conducted using cDNA and RNA after DNase treatment as the template. RNA abundances were only taken into account if the copy numbers in the RNA samples were around the detection limit and at least 100× lower than the copy numbers in the cDNA samples.

## Supplementary Materials

Supplementary materials can be accessed at: http://www.mdpi.com/2075-1729/5/2/1396/s1.
