# Supplementary material for: Freshwater Ammonia-Oxidizing Archaea Retain amoA mRNA and 16S rRNA during Ammonia Starvation
Source: Life (Basel). 2015 May 19;5(2):1396–404. doi: 10.3390/life5021396 (PMC4500144; doi:10.3390/life5021396)
Supplement: Supplementary file 1 [file life-05-01396-s001.pdf]

Supplementary Materials

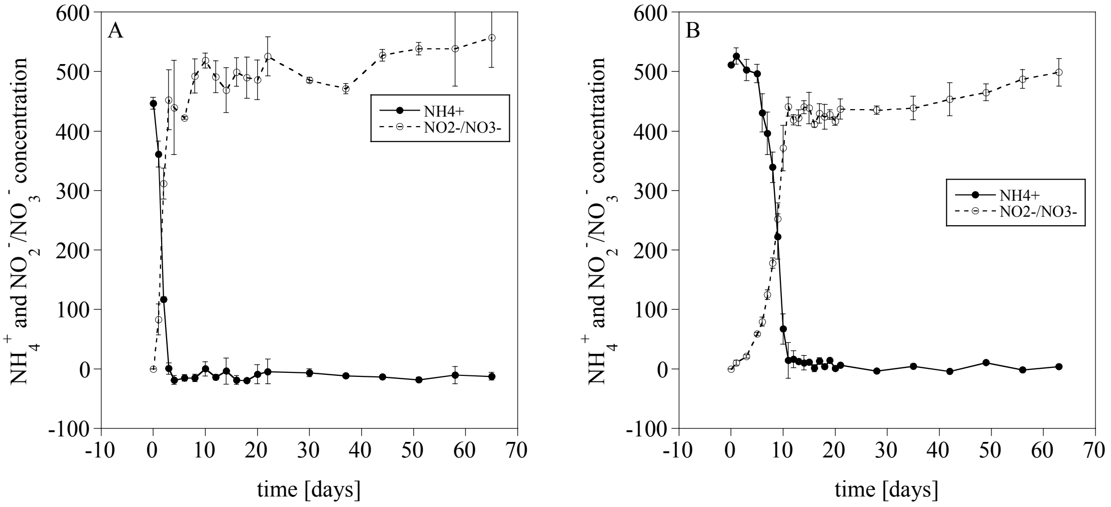

Figure S1. Ammonium consumption and nitrite/nitrate production over time in the starvation cultures of AOA-AC1 and AOB-G5-7 (mean ± SD, *n* = 3). Starvation started when ammonium was consumed and completely converted to nitrite/nitrate.

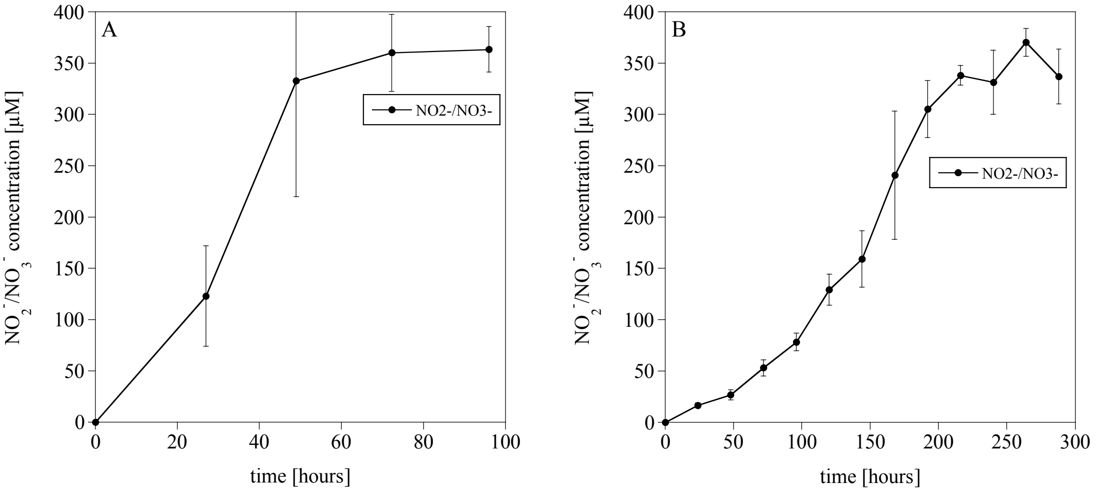

Figure S2. Nitrite/nitrate production over time in the example recovery cultures of AOA-AC1 and AOB-G5-7 (mean ± SD, *n* = 3).

Table S1. Specific growth rates [h^{-1}] of the recovery cultures after starvation of the AOB enrichment culture AOB-G5-7 and the AOA enrichment culture AOA-AC1 (data are similar to Figures 1 and 2) (mean ± SD, n = 3; different letters behind values indicate significant differences between values determined by one-way ANOVA followed by Tukey test; $p < 0.05$). Starvation started at day 0.

Starvation Time [Days]	AOB-G5-7	AOA-AC1
−1	0.0461 ± 0.0038 ^{a}	0.0201 ± 0.0016 ^{a}
0		0.0171 ± 0.0008 ^{ab}
1	0.0504 ± 0.0031 ^{a}	0.0170 ± 0.0007 ^{ab}
2		0.0182 ± 0.0023 ^{ab}
3	0.0463 ± 0.0013 ^{a}	0.0162 ± 0.0006 ^{ab}
4		0.0165 ± 0.0002 ^{ab}
5	0.0509 ± 0.0032 ^{a}	0.0163 ± 0.0017 ^{ab}
9	0.0525 ± 0.0137 ^{a}	0.0147 ± 0.0005 ^{bc}
11	0.0433 ± 0.0060 ^{a}	
13	0.0492 ± 0.0169 ^{a}	
15	0.0445 ± 0.0014 ^{a}	
16		0.0120 ± 0.0005 ^{cd}
19	0.0436 ± 0.0012 ^{a}	
23		0.0112 ± 0.0027 ^{cd}
24		
26	0.0472 ± 0.0084 ^{a}	
30		0.0107 ± 0.0013 ^{d}
33	0.0523 ± 0.0040 ^{a}	
37		0.0121 ± 0.0004 ^{cd}
40	0.0523 ± 0.0035 ^{a}	
44		0.0105 ± 0.0009 ^{d}
47	0.0513 ± 0.0031 ^{a}	
52		0.0118 ± 0.0004 ^{cd}

Table S2. Lag phase [h] of the recovery cultures after starvation of the AOB enrichment culture AOB-G5-7 and the AOA enrichment culture AOA-AC1 (data are similar to Figure 1) (mean ± SD, $n = 3$; different letters behind values indicate significant differences between values determined by one-way ANOVA followed by Tukey test; $p < 0.05$). Starvation started at day 0.

Starvation Time [Days]	AOB-G5-7	AOA-AC1
−1	24 ± 0 [a]	24 ± 0 [a]
0		32 ± 13.9 [a]
1	24 ± 0 [a]	40 ± 13.9 [a]
2		24 ± 0 [a]
3	24 ± 0 [a]	32 ± 13.9 [a]
4		32 ± 13.9 [a]
5	24 ± 0 [a]	32 ± 13.9 [a]
7	24 ± 0 [a]	40 ± 13.9 [a]
9	24 ± 0 [a]	72 ± 0 [ab]
11	24 ± 0 [a]	
13	24 ± 0 [a]	
15	24 ± 0 [a]	
16		128 ± 13.9 [bc]
19	24 ± 0 [a]	
23		152 ± 99.9 [bc]
26	24 ± 0 [a]	
30		152 ± 13.9 [bc]
33	24 ± 0 [a]	
37		160 ± 13.9 [c]
38		
40	24 ± 0 [a]	
44		144 ± 0 [bc]
47	24 ± 0 [a]	
51		144 ± 0 [bc]

Table S3. Influence of starvation time [days] on RNA concentration [ng/µl] in the enrichment cultures AOB-G5-7 and AOA-AC1 (mean ± SD, $n = 3$; different letters behind values indicate significant differences between values determined by one-way ANOVA of the log-transformed copy numbers followed by Tukey test; $p < 0.05$). Starvation started at day 0.

Starvation Time [Days]	AOB-G5-7	AOA-AC1
−1	18.8 ± 1.7 [a]	91.2 ± 5.4 [a]
1	22.8 ± 1.3 [a]	108.3 ± 13.6 [a]
9		91.7 ± 29.2 [a]
19	28.4 ± 4.1 [ab]	
23		72.6 ± 8.5 [a]
34	28.2 ± 2.5 [ab]	
44		82.4 ± 9.7 [a]
48	36.8 ± 6.9 [b]	

Table S4. Influence of starvation time [days] on *amoA* copy number [copies/ng RNA] of the enrichment cultures AOB-G5-7 and AOA-AC1 (data are similar to Figures 3 and 4) (mean ± SD, $n = 3$; different letters behind values indicate significant differences between values determined by one-way ANOVA of the log-transformed copy numbers followed by Tukey test; $p < 0.05$). Starvation started at day 0.

Starvation Time [Days]	AOB-G5-7	AOA-AC1
−1	$2.77 \times 10^5 \pm 0.08 \times 10^5$ [a]	$6.02 \times 10^5 \pm 0.59 \times 10^5$ [a]
1	24119 ± 2119 [b]	13945 ± 8158 [b]
10		5952 ± 2555 [bc]
18		4699 ± 2333 [bc]
25		2193 ± 588 [c]
46		2441 ± 922 [c]

Table S5. Influence of starvation time [days] on 16S rRNA copy number [copies/ng RNA] of the enrichment cultures AOB-G5-7 and AOA-AC1 (data are similar to Figures 3 and 4) (mean ± SD, $n = 3$; different letters behind values indicate significant differences between values determined by one-way ANOVA of the log-transformed copy numbers followed by Tukey test; $P < 0.05$). Starvation started at day 0.

Starvation Time [Days]	AOB-G5-7	AOA-AC1
−1	$1.27 \times 10^8 \pm 0.15 \times 10^8$ [a]	$4.04 \times 10^7 \pm 0.56 \times 10^7$ [a]
1	$1.09 \times 10^8 \pm 0.03 \times 10^8$ [a]	$4.85 \times 10^7 \pm 2.06 \times 10^7$ [a]
10		$4.01 \times 10^7 \pm 1.27 \times 10^7$ [a]
16	$0.71 \times 10^8 \pm 0.20 \times 10^8$ [a]	
18		$5.82 \times 10^7 \pm 1.21 \times 10^7$ [a]
25		$4.28 \times 10^7 \pm 2.31 \times 10^7$ [a]
32	$0.58 \times 10^8 \pm 0.46 \times 10^8$ [a]	
46	$0.66 \times 10^8 \pm 0.14 \times 10^8$ [a]	$2.87 \times 10^7 \pm 0.88 \times 10^7$ [a]

Table S6. Influence of starvation time [days] on eubacterial 16S rRNA copy number of the enrichment cultures AOB-G5-7 and AOA-AC1 (data are similar to Figures 3 and 4) (mean ± SD, $n = 3$; different letters behind values indicate significant differences between values determined by one-way ANOVA of the log-transformed copy numbers followed by Tukey test; $P < 0.05$). Starvation started at day 0.

Starvation Time [Days]	AOB-G5-7	AOA-AC1
−1	$1.02 \times 10^8 \pm 0.13 \times 10^8$ [a]	$1.10 \times 10^7 \pm 0.15 \times 10^7$ [a]
1	$1.09 \times 10^8 \pm 0.06 \times 10^8$ [a]	$0.99 \times 10^7 \pm 0.43 \times 10^7$ [a]
10		$0.83 \times 10^7 \pm 0.34 \times 10^7$ [a]
16	$0.69 \times 10^8 \pm 0.34 \times 10^8$ [a]	
18		$1.18 \times 10^7 \pm 0.03 \times 10^7$ [a]
25		$0.97 \times 10^7 \pm 0.19 \times 10^7$ [a]
32	$0.77 \times 10^8 \pm 0.04 \times 10^8$ [a]	
46	$0.70 \times 10^8 \pm 0.10 \times 10^8$ [a]	$1.37 \times 10^7 \pm 0.19 \times 10^7$ [a]

Table S7. Primers used quantification of *amoA* and 16S rRNA genes.

	**Primer**
AOA-AC1 *amoA*	140F: 5’-GTA GTC GGC GCA TGC TAC T-3’<br>244R: 5’-CCA TGC ACC TTT TGC TAC CC-3’
AOA-AC1 16S rRNA	398F: 5’-TCC GAG TGT CTT CTG CTA AG-3’<br>549R: 5’-CCC AAT AAA CCT CCT GAC CA-3’
AOB-G5-7 *amoA*	415F: 5’-CTG TTG ACG GGT AAC TGG CT-3’<br>514R: 5’-AGT GGG TCG GGC CAA ATA TC-3’
AOB-G5-7 16S rRNA [25]	189C-F: 5’-GGA GGA AAG TAG GGG ATC G-3’<br>295R: 5’-GAC CAA CTA CTG ATC GTT GCC-3’
Eubacterial primers [26]	357F: 5’-CCT ACG GGA GGC AGC AG-3’<br>518R: 5’-ATT ACC GCG GCT GCT GG-3’

Table S8. PCR conditions (temperature [°C]/time [s]) and validation of qPCR.

	**AOA *amoA***	**AOA 16S**	**AOB *amoA***	**AOB 16S**	**Eubac**
Denaturation (initial)	95/600	95/600	95/600	95/600	95/600
Denaturation	95/15	95/15	95/15	95/15	95/15
Annealing	56/30	57/30	56/30	57/30	61/30
Cycles	35	35	35	35	30
Melting curve	95	95	95	95	95
	55	55	55	55	55
Efficiency [%]	102	94	104	96	98–99
R^2	0.99	0.99	0.99	0.99	0.99
Concentration for calibration curve	10^2–10^6	10^3–10^7	10^2–10^6	10^3–10^7	10^3–10^7
